# The prevalence of rheumatoid arthritis in middle-aged and elderly people living in Naqu City, Tibet, Autonomous Region of China

**DOI:** 10.1186/s13018-020-01883-4

**Published:** 2020-08-18

**Authors:** Qingxi Zhang, Qiang Liu, Chutong Lin, Yangjin Baima, Hu Li, Hongqiang Gong, Jianhao Lin

**Affiliations:** 1 Arthritis Clinic & Research Center, Peking University People’s Hospital, Peking University, 11th Xizhimen South Street, Beijing, China; 2 Arthritis Institute, Peking University, Beijing, China; 3 Department of Rheumatism, Tibet Autonomous Region People’s Hospital, Tibet, China; 4 Tibet Center for Disease Control and Prevention, No. 21, North Linkuo Road, Chengguan District, Lhasa City, 850000 Tibet Autonomous Region Tibet China

**Keywords:** Rheumatoid arthritis, Tibet, High altitude

## Abstract

**Objective:**

To estimate the prevalence of rheumatoid arthritis (RA) in the Tibet Autonomous Region (China).

**Methods:**

A population-based cross-sectional survey was conducted on 1458 residents of Luoma Town, Tibet Autonomous Region, who were aged ≥ 40 years old. We interviewed participants using questionnaires, and rheumatoid factor (RF), anti-citrullinated protein antibodies (ACPA), and C-reactive protein (CRP) were determined. The identification of RA in this study was on the basis of criteria issued by the 2010 American College of Rheumatology/European League Against Rheumatism (ACR/EULAR) guideline.

**Results:**

Herein, 782 participants completed all items of RA. The overall prevalence of RA was 4.86%, and the prevalence was higher in women than that in men (7.14% vs. 2.56%, *p* = 0.005). The age-standardized prevalence of RA was 6.30% (95% confidence interval (CI) 4.20–8.64%), which was 2.46% (95% CI 1.04%, 4.10%) and 9.59% (95% CI 5.93%, 13.77%) in men and women, respectively.

**Conclusion:**

The prevalence of RA is relatively higher in the Tibet than that in other areas of China.

## Introduction

Rheumatoid arthritis (RA) is a chronic systematic autoimmune inflammatory disease characterized by a symmetrical inflammatory polyarthritis of the joints of the hands, wrist, feet, and knee, which may result in joint swelling, pain, stiffness, and possible loss of function [1]. It seriously affects the life quality of patients. As reported previously, women are at a higher risk of RA than men, with the highest prevalence among women aged over 65 years [2]. Almost 20 million people were living with RA in 2017 globally, with over one million new cases diagnosed each year, according to estimates from the Global Burden of Diseases, Injuries, and Risk Factors (GBD) Study [3]. However, due to the different geographic and demographic factors, the prevalence of RA is different in a variety of regions. The disease is more common in northern Europe and North America, while it is scarcely reported in the developing countries [4].

In China, the first epidemiological study on RA was not reported until 1983, with a prevalence of 0.3% in Taiwan [5]. Several epidemiological researches conducted across China mainland were published, and it was shown that the prevalence of RA ranges from 0.2 to 0.93% in China [6]. However, China is a vast country with gradual altitude decreasing from the western to the eastern areas [7]. To our knowledge, there are few concerns about the prevalence of RA in Western China, especially Tibet. Since the 18th National Congress of the Communist Party of China, the alleviation of poverty in China has made a remarkable progress. Medical aid for Tibet Autonomous Region is of great significance. The People’s Republic of China gives top priority to the improvement of Tibetan’s livelihoods. However, collection of epidemic data and making clinical decisions are very difficult due to the widely distributed population of Tibet across high mountain areas [8]. Therefore, conducting further study is essential and urgent with the aim of describing the prevalence of RA in Tibet area.

## Subjects and methods

### Subjects

This was a cross-sectional survey and the target population was participants who were aged ≥ 40 years old and were residents of the Tibet. Luoma Town was randomly selected from 12 towns in Seni Community, Naqu City, Tibet, China. Luoma Town was located in northern Tibet with an average altitude above 4500 m. In the current study, first, one community was randomly selected from Naqu City. Next, one town was randomly selected from the former community. The cluster (Luoma Town) covered 12 villages, and all households in the cluster were included in this study. All inhabitants, who were aged ≥ 40 years old and were self-described residents of Luoma Town, were included. Individuals who had disability, mental disorders, and malignant tumors, and lived away from home for more than half a year were excluded from the survey. The survey was approved by the Ethics Committee of Peking University Health Science Center (Beijing, China). Written informed consent was obtained from all participants, and the survey was conducted in accordance with the Declaration of Helsinki.

### Questionnaire

The questionnaire was administered to health care professionals, as it was anticipated that several participants would be illiterate and could not speak mandarin. All interviewers and clinical examiners were trained under the supervision of principal investigators (Hongqiang Gong and Jianhao Lin). Sociodemographic characteristics (e.g., sex, age, levels of education) were queried.

### Clinical and laboratory examinations and definition of RA

Height was measured with a wall-mounted stadiometer using the average of two measurements taken. Body weight was assessed using a beam balance scale with 0.1 kg precision. Two professionals took history. Venous blood was collected from each consented respondent by medically trained staffs from the Chinese Center for Disease Control and Prevention based on a standard protocol. The centrifuged blood samples were stored in a deep freezer in the local health station. After samples were fully collected, the blood-based bioassays were performed at Tibet Autonomous Region People’s Hospital. We tested the rheumatoid factor (RF), anti-citrullinated protein antibodies (ACPA), and C-reactive protein (CRP). The RF and CRP levels were detected using turbidimetric inhibition immune-assay, and ACPA was determined using chemiluminescent immunoassay. RA was identified according to the criteria issued by the 2010 American College of Rheumatology/European League Against Rheumatism (ACR/EULAR) guideline [9].

### Statistical analysis

Quantitative data were analyzed using EpiData software (EpiData Association, Odense, Denmark). We divided all subjects into four age-based groups (i.e., 40–49, 50–59, 60–69, and ≥ 70 years old). The levels of education were classified into elementary school and below, middle school, high school, and college and higher. All statistical analyses were performed using SPSS 22.0 software (IBM, Armonk, NY, USA). Continuous variables and categorical variables were expressed as mean ± standard deviation (SD) and proportions, respectively. According to Chinese census population in 2010, the national average age was adopted as the standard and the direct method was used to calculate the standardized mortality rates in various conditions [10]. Quantitative variables were summarized as mean values and 95% confidence interval (CI). Differences between groups were analyzed by chi-square test or Fisher’s exact test for categorical data, if appropriate. Univariate and multivariate logistic regression analyses were undertaken to estimate the odds ratio (OR) and CI of factors associated with RA occurrence. *p* < 0.05 was considered statistically significant.

## Results

### Participants

**Fig. 1:**
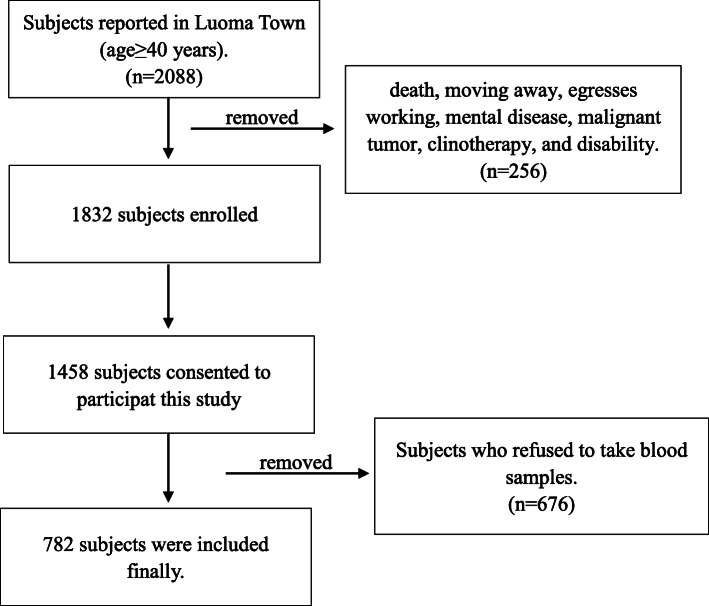
Flowchart of subjects screening

In this survey, 2088 subjects were recruited, who were aged ≥ 40 years old, and 256 subjects were excluded due to aforementioned reasons. Finally, 1832 subjects were enrolled, and 1458(80%)subjects signed the written informed consent form and completed the survey between September and October 2018. The attended participants were younger than those who declined to participate (52.30 ± 8.43 versus 58.5 ± 3.42, *p* = 0.106). Moreover, 782 participants completed all items of RA and attended in laboratory tests (Fig. 1). The sociodemographic characteristics of eligible participants are shown in Table 1. There was no significant difference between participants who took blood samples and those who refused to take blood samples, except for age-relevant characteristics. About half (50.13%) of the included subjects were women, and their mean age (52.79 ± 8.74 years old) was almost equal to the mean age of men (51.81 ± 8.07 years old). For levels of education, the majority of men and women only received elementary education (97.70%). The mean BMI of women was greater than that of men, while overweight was indicated for both women and men.

**Table 1:** Sociodemographic characteristics of the subjects

Subjects who consent to take blood samples				Subjects who refused to take blood samples		
	Male 49.87% (*n* = 390)	Female 50.13%(*n* = 392)	Total(782)	Male 48.52%(*n* = 328)	Female 51.48%(*n* = 348)	Total(676)
Age, mean ± SD	51.81 ± 8.07	52.79 ± 8.74	52.30 ± 8.43	54.17 ± 9.79	53.47 ± 10.24	53.81 ± 10.0*
Age group, % (*n*)						
40–50	41.87%(164)	45.13%(176)	43.48%(340)	38.41%(126)	44.54%(155)	41.57%(281)
50–59	36.99%(145)	38.21%(149)	37.60%(294)	34.45%(113)	29.89%(104)	32.1%(217)
60–69	16.58%(65)	13.33%(52)	14.96%(117)	19.51%(64)	16.67%(58)	18.05%(122)
≥ 70	4.59%(18)	3.33%(13)	3.96%(31)	7.62%(25)	8.91%(31)	8.28%(56)
Ethnicity,% (*n*)						
Tibetan	99.74%(389)	99.74%(391)	99.74%(780)	100%(328)	100%(348)	1000%(676)
Moinba	0%(0)	0.26%(1)	0.10%(1)	0%(0)	0%(0)	0%(0)
Others	0.26%(1)	0%(0)	0.10%(1)	0%(0)	0%(0)	0%(0)
Education,% (*n*)						
Elementary education and below	98.46%(384)	99.23%(389)	97.70%(763)	48.60%(312)	50.62%(325)	99.22%(637)
Middle/high school	1.28%(5)	0.51%(2)	0.90%(7)	0.62%(4)	0.16%(1)	0.78%(5)
College and higher	0%(0)	0.26%(1)	0.13%(1)	0%(0)	0%(0)	0%(0)
BMI, mean ± SD	25.25 ± 7.02	26.32 ± 17.31	25.82 ± 4.36	25.13 ± 11.63	24.45 ± 4.50	24.78 ± 8.85

*BMI* body mass index

*p < 0.05

### The prevalence of RA in Tibet

**Fig. 2:**
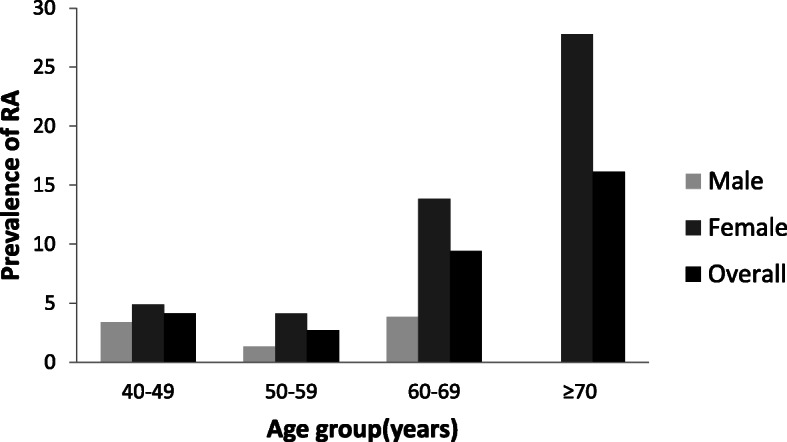
The prevalence of rheumatoid arthritis (RA) in four age groups

The overall prevalence of RA was 4.86%, and it was more prevalent in women than that in men (7.14% vs. 2.56%, *p* = 0.005). The age-standardized prevalence of RA was 6.30% (95% CI 4.20–8.64%), which was 2.46%(95% CI 1.04%, 4.10%)and 9.59% (95% CI 5.93%, 13.77%) in men and women, respectively, as shown in Table 2. Meanwhile, the prevalence of women increased with age (Fig. 2). The association of each factor (age, ethnicity, levels of education, and obesity) with the occurrence of RA was analyzed; however, no significant difference was noted.

**Table 2:** The prevalence of rheumatoid arthritis

The prevalence of rheumatoid arthritis				
	Male	Female		Overall
Crude, %	2.56% (2.318%, 2.82%)	7.14% (6.44%, 7.85%)	*p* = 0.005	4.86% (4.52%, 5.20%)
Age-standardized, %	2.46% (1.04%, 4.10%)	9.59% (5.93%, 13.77%)		6.30% (4.20%, 8.64%)

## Discussion

The current cross-sectional study involved a regional representative sample of the middle-aged and older Tibet inhabitants, and the overall prevalence of RA was estimated to be 6.30%. Although there was a significant difference in age between those who refused to take blood test and those who underwent blood test, the mean age of the former was higher than the latter. Additionally, the prevalence of RA increased with age; therefore, the true prevalence may be higher. This amazing rate, which is close to the highest prevalence of RA (up to 6.80%) worldwide [11], is remarkably different from a previously reported rate (0.28%) in China [12]. The prevalence in the group of ≥ 45 years old was remarkably lower than that of the current study (0.74%). Similarly, the prevalence in both men (2.46%) and women (9.59%) in our research was higher than that in a previous study (0.19% in men and 1.28% in women, aged > 45 years old) [12]. However, the prevalence of RA in women increased with age, which reached the peak after 60 years old, and that was consistent with previous researches [6, 12]. A study showed that the prevalence of RA significantly varies geographically [13]. As China is geographically a large country with a multi-ethnic population and substantial regional differences in socio-economic and hygienic conditions, the result may not represent China as a whole [14]. Since 1983, a great number of studies have been performed in China to investigate the epidemiologic characteristics of RA, with concentration on differences among different regions [5, 15–25]. In those studies, the prevalence of RA ranged from 0.2% (Shantou) to 0.93% (Taiwan), which was remarkably lower than our results. However, the main concern of those researches was the prevalence in low altitude areas of the east-central China, and the majority of the participants were Han nationality [26].

A number of scholars pointed out that the prevalence of RA differs in different regions of the world, which indicated that the etiology of this disease could be influenced by both genetic and environmental factors [27]. Tibet is known as the “Third Pole” and is one of the inhabited areas in the globe, highlighting that the geographical condition of Tibet is different from the inland or coastal areas of China [28]. A research pointed out that the people living at high altitudes showed microflora enriched with butyrate-producing bacteria in response to harsh environments, and core microbiota comprised of Prevotella, Faecalibacterium, and Blautia in Tibetans [29]. Moreover, it was reported that the presence of Prevotella is strongly correlated with disease in new-onset untreated rheumatoid arthritis (NORA) patients [30]. Therefore, this may justify the high prevalence of RA in Tibetans. It is noteworthy that almost all the participants examined in the present study were Tibetans (99.74%) living in Luoma Town. In addition, Tibet is an isolated area in mainland China, and the highlanders have lived there for generations, which mean they have distinct genetic backgrounds with a lower level of heterozygosity, and a higher level of runs of homozygosity [31]. To our knowledge, 50% of the risk of RA is attributable to genetic factors [1]. This may explain a remarkably higher prevalence of RA in Tibet compared with that in other areas of China. The prevalence of RA of native American Chippewa Band (6.8%) is far above the other Americans (1.07%) [12, 32]. This is not a coincidence, but further study is needed to confirm it.

Our results demonstrated that the prevalence of RA reaches the highest in high altitude areas of Tibet. Therefore, further attention should be paid to the prevention and treatment of RA in Tibet.

### Strengths and limitations

This is the first cross-sectional study concentrating on spreading of RA in the Tibet Autonomous Region. Our results may provide a reliable reference for future research on RA in Tibet. However, this study contains a number of limitations. Firstly, selection bias may exist because of subjects who refused to take blood samples, and that factor may influence our results. Secondly, although it is concluded that the prevalence of RA is extremely high in Tibetans, we did not assess the mechanism and cause of RA, which is worthy of further investigation.

## Conclusions

This is the first cross-sectional study concentrating on RA status in the Tibet Autonomous Region. Our findings unveiled that the prevalence of RA in Tibetans was the highest in mainland China. Future studies should further investigate the roles of genetic and environmental factors. Besides, further attention should be paid on behalf of the central government of the People’s Republic of China to treat RA in the Tibet.

## Data Availability

The first author can provide all data.
